# Raising the avermectins production in *Streptomyces avermitilis* by utilizing nanosecond pulsed electric fields (nsPEFs)

**DOI:** 10.1038/srep25949

**Published:** 2016-05-16

**Authors:** Jinsong Guo, Ruonan Ma, Bo Su, Yinglong Li, Jue Zhang, Jing Fang

**Affiliations:** 1 College of Engineering, Peking University, Beijing, 100871, China; 2 Academy for Advanced Interdisciplinary Studies, Peking University, Beijing, 100871, China

## Abstract

Avermectins, a group of anthelmintic and insecticidal agents produced from *Streptomyces avermitilis*, are widely used in agricultural, veterinary, and medical fields. This study presents the first report on the potential of using nanosecond pulsed electric fields (nsPEFs) to improve avermectin production in *S. avermitilis*. The results of colony forming units showed that 20 pulses of nsPEFs at 10 kV/cm and 20 kV/cm had a significant effect on proliferation, while 100 pulses of nsPEFs at 30 kV/cm exhibited an obvious effect on inhibition of agents. Ultraviolet spectrophotometry assay revealed that 20 pulses of nsPEFs at 15 kV/cm increased avermectin production by 42% and reduced the time for reaching a plateau in fermentation process from 7 days to 5 days. In addition, the decreased oxidation reduction potential (ORP) and increased temperature of nsPEFs-treated liquid were evidenced to be closely associated with the improved cell growth and fermentation efficiency of avermectins in *S. avermitilis*. More importantly, the real-time RT-PCR analysis showed that nsPEFs could remarkably enhance the expression of *aveR* and *malE* in *S. avermitilis* during fermentation, which are positive regulator for avermectin biosynthesis. Therefore, the nsPEFs technology presents an alternative strategy to be developed to increase avermectin output in fermentation industry.

Avermectins and its analogs, produced from a gram-positive bacterium named *Streptomyces avermitilis*, are major commercial antiparasitic agents applied in agriculture, animal health and human anti-infection^1^. There are eight major avermectin components (A1a, A2a, A1b, A2b, B1a, B2a, B1b and B2b) resulting from structural differences at C5, C22-C23, and C26. Among those compounds, the B1a component has the most effective antiparasitic activity^2^. Avermectins are a novel class of 16-membered ring macrolide antibiotics, not only exhibiting excellent anthelmintic activity against nematode and arthropod parasites, but having low toxic side effects on the host organism as well^2^,^3^. On the other hand, raising the production of avermectins is a big challenge with commercial importance in the biological pesticide market, because its output is remarkably insufficient for human consumption nowadays.

Basically, there are three conventional approaches to improve the fermentation efficiency of avermectins in *S. avermitilis*: utilization of mutagens (such as ultraviolet, atmospheric pressure cold plasma, lithium chloride and nitrosoguanidine)^4^,^5^, genetic engineering technology^6^,^7^, and optimization of fermentation conditions^8^,^9^. In fact, each technique has its disadvantages. For the using of mutagens, positive mutants are produced accompanied by the formation of negative mutants^5^,^10^. Referring to genetic engineering technology, the majority of bacterial recombinant strains bearing genes cloned in plasmids are known to be often unstable both in batch and prolonged continuous cultivation in a chemostat^11^. With respect to optimizing the fermentation conditions, it has limited capacity to improve the fermentation efficiency^10^,^12^. Therefore, an alternative effective and easy-to-apply technology to yield amount of avermectins in *S. avermitilis* has been taken on a high priority.

Recently, nanosecond pulsed electric fields (nsPEFs) is emerging as a novel non-thermal technology concerned in the biomedical field, including electro-gene therapy^13^, cell electrofusion^14^, tumor therapy^15^,^16^,^17^, bacteria inactivation^18^, platelet gel formation^19^, calcium mobilization^20^ and alteration of cell membrane permeability^21^. Unlike conventional electroporation, nsPEFs exhibits extremely short pulse durations (nanoseconds), high electric fields (kV/cm), but low energy (mJ/cc) and non-thermal effects^22^. More recently, an interest is growing in concerning the proliferation effect induced by nsPEFs under relatively low electric field strength. Several researches have been reported that nsPEFs could improve the growth of *Arabidopsis thaliana*^23^,^24^ and *Haloxylon ammodendron* seeds^25^ as well as enhance the proliferation and dedifferentiation of chondrocytes^26^. Inspired by the promising capability of nsPEFs for cell proliferation enhancement, we attempt to introduce this pulsed power technology to stimulate cell growth and to increase avermectins production of *S. avermitilis*.

In this study, nsPEFs was applied to treat the spore suspensions of *S. avermitilis*. The cell viability of *S. avermitilis* after treatment was examined via colony forming units (CFU) count, and the morphology change of *S. avermitilis* was detected by scanning electron microscope (SEM). The cell growth curve and the total avermectins production of *S. avermitilis* were measured by UV spectrometer at 450 nm and 245 nm, respectively. Furthermore, the changes in the expression of three key genes (*aveR, malE* and *σ*^25^) related to the avermectins production during the fermentation were analyzed by real-time RT-PCR. To better understand the mechanism of nsPEFs induced *S. avermitilis* proliferation enhancement, the physicochemical properties of nsPEFs-treated phosphate buffer saline (PBS) without spore were evaluated and compared by recording the oxidation reduction potential (ORP), electrical conductivity, pH and temperature.

## Results and Discussions

### nsPEFs device and the proposed experimental plan

**Figure 1:**
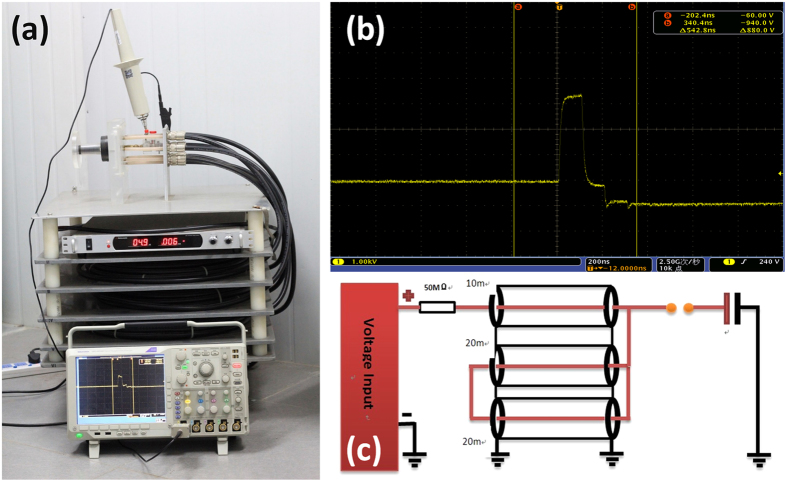
The pulse generator used in experiment. (**a**) Schematic of the experimental apparatus for nsPEFs on *S. avermitilis*. (**b**) The typical oscillogram of 100 ns pulse generator. (**c**) Circuit diagram of the basic Blumlein pulse forming system.

As shown in Fig. 1, a self-made nsPEFs power generator was established on the basis of transmission line circuit with a pulse duration of 100 ns as previously described^13^, while the electric fields varied from 10 kV/cm to 60 kV/cm. The pulse waveforms were monitored using a digital phosphor oscilloscope (DPO4054, Tektronix, USA) equipped with a high voltage probe (P6015A, Tektronix, USA). The *S. avermitilis* spore suspensions were placed in 0.4 cm gap cuvette (Biosmith, aluminum plate electrodes) and then exposed to nsPEFs.

**Figure 2:**
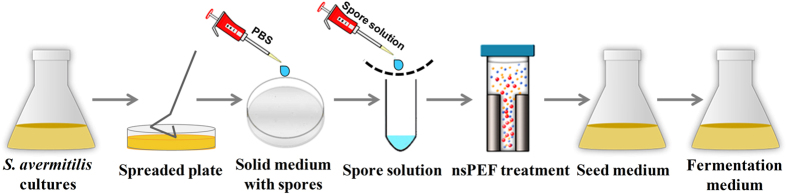
A schematic diagram of the experimental arrangement, including preparation of *S. avermitilis* spore suspension, nsPEF treatment and fermentation of *S. avermitilis*.

The experimental protocol for nsPEFs treatment of *S. avermitilis* spores is shown in Fig. 2. The cell suspension of *S. avermitilis* cultured in their vegetative state was spread onto solid YMS medium, and then grew in a batch culture for 7–10 d until the transition to spores exceeded 95%. The spores were then removed and rinsed with 10 ml sterilized PBS (pH 7.0) solution to form spore suspension, which was then filtered by 4-layer sterilized gauze to remove *S. avermitilis* mycelia. The spore suspensions (1 ml) were treated by nsPEFs with different electric fields, while the spore suspensions without any treatment were used as the control. After nsPEFs treatment, the spores were collected, washed twice with PBS and resuspended in 1 ml PBS. Then, 1 ml spore suspensions were cultured in 50-ml Erlenmeyer flasks containing 20 ml seed medium, with glass beads, for 24 h at 28 °C on a rotary shaker (180 rpm). A 2-ml sample of the seed culture was inoculated into a 250-ml flask containing 100 ml fermentation medium. The cells were cultured for 9–11 days at 28 °C with shaking (220 rpm) to examine the yield of avermectins.

### The cell viability of *S. avermitilis*

**Figure 3:**
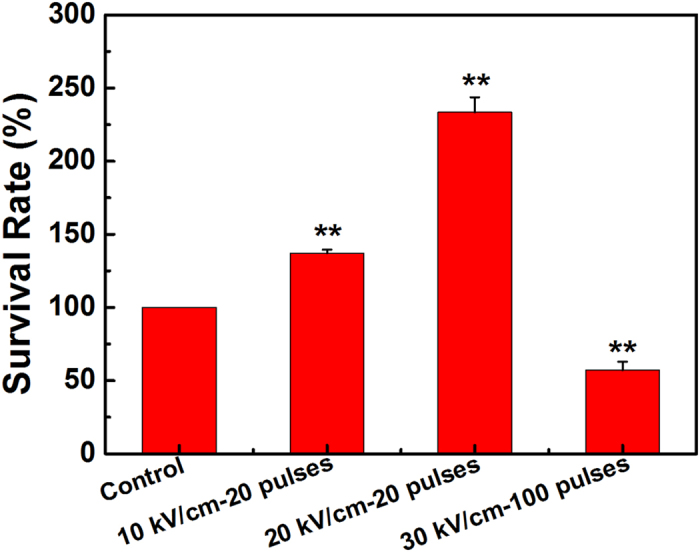
Survival rate of *S. avermitilis* without (control) and with nsPEFs treatment of 20 pulses at 10 and 20 kV/cm, and 100 pulses at 30 kV/cm. Values are the mean ± standard deviation of three independent biological replicate experiments (n = 3). Statistical significance of differences between the nsPEFs-treated and control groups is indicated with **when *P* < 0.01.

The *S. avermitilis* spore suspensions were exposed to 20 pulses of nsPEFs at 10 and 20 kV/cm, as well as 100 pulses of nsPEFs at 30 kV/cm, respectively. And the spore suspension without any treatment served as the control. The cell viability of *S. avermitilis* was evaluated to determine the optimum dose of nsPEFs treatment for *S. avermitilis* producing avermectin through CFU assay. The results in Fig. 3 show that the survival rate was 137% after 20 pulses of nsPEFs at 10 kV/cm, and proliferation rate burst to 233% for that treated by 20 pulses at 20 kV/cm. However, as the electric field intensity and the pulse number increased to 30 kV/cm and 100 pulses, the survival rate dropped to 57%. It indicates that the nsPEFs treatment with low electric field strength and less pulse number results in cell proliferation of *S. avermitilis*, whereas the treatment with high electric field strength and more pulse number exhibits obviously inhibition effect. This result is consistent with our previous report of nsPEFs enhancing cell proliferation of chondrocytes, in which the nsPEFs at 10 kV/cm presented a slight increase of proliferation while that cell proliferation was significantly increased at 20 kV/cm^26^. This point can also be evidenced from the work of B. Su *et al*. on seed growing, where the nsPEFs treatment with low intensity could elevate the germination rate of *Haloxylon ammodendron* seed, while high field intensity would lead to significant inhibition^25^.

**Figure 4:**
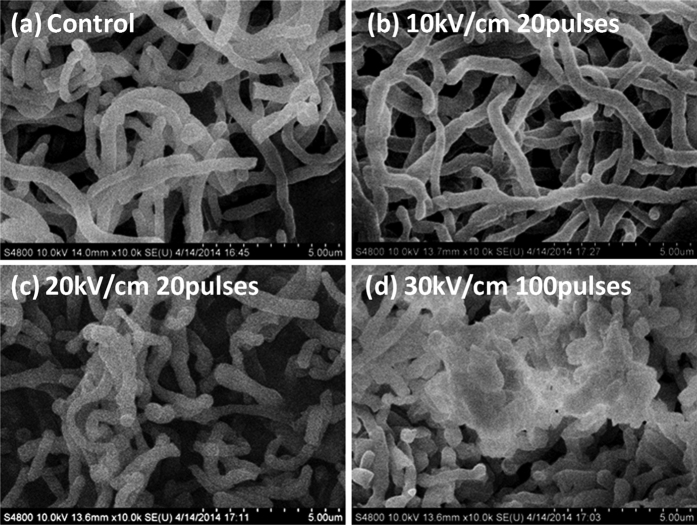
SEM pictures of *S. avermitilis* at 10000-magnification: (**a**) the control group without nsPEFs treatment, (**b**) the spores treated by 10 kV/cm nsPEFs with 20 pulses, (**c**) by 20 kV/cm nsPEFs with 20 pulses, (**d**) and by 30 kV/cm nsPEFs with 100 pulses.

The SEM pictures of *S. avermitilis* shown in Fig. 4 include the control, those after nsPEFs exposures of 20 pulses at 10 kV/cm and 20 kV/cm, whose surfaces are intact and smooth (Fig. 4a–c) On the other hand, the surface morphology changes significantly for the bacteria processed by 100 pulses of nsPEFs at 30 kV/cm, exhibiting obvious distortion and shrinkage of the outer layer (Fig. 4d). These different levels of morphological damage support the results of survival rate of *S. avermitilis* above and also prove the effects of nsPEFs on proliferation or inhibition of the cells with dependence on the electric field intensity and pulse number. Based on above results, in the following experiments we set up the e nsPEFs parameters as 5, 10 and 15 kV/cm with 20 pulses to obtain satisfied cell proliferation of *S. avermitilis*.

### Effect of nsPEFs on cell growth of *S. avermitilis*

**Figure 5:**
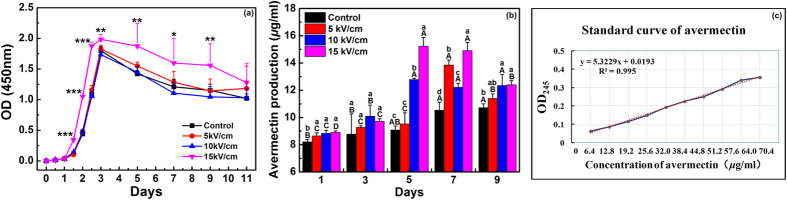
Cell growth of *S. avermitilis* during 11-day culture and avermectin productivity of *S. avermitilis* during 9-day fermentation. (**a**) Cell growth of *S. avermitilis* without (control) and with nsPEFs treatment in soluble FM-II during 11-day culture. Values are the mean ± standard deviation of three independent biological replicate experiments (n = 3). Statistical significance of differences between nsPEFs-treated and control cells is indicated with *when *P* < 0.05, **when *P* < 0.01. (**b**) Overall avermectin production of *S. avermitilis* without (control) and with nsPEFs treatment cultured in fermentation medium for 9 days. Vertical bars represent the mean ± standard deviation of three independent biological replicate experiments (n = 3). Bars labeled with different lowercase letters within the same fermentation day indicate a significant difference (*P* ≤ 0.05) and different uppercase letters within the same treatment indicate a significant difference (*P* ≤ 0.05). (**c**) The standard curve of avermectins.

To evaluate the effects of nsPEFs on the cell growth of *S. avermitilis* in the long term, OD_450_ were recorded every two days during an 11-day culture to obtain their cell growth curves^27^. As shown in Fig. 5a, the cell growth curve of control (black) presents an abbreviated S-shaped logistic pattern, meaning that the cells proliferated exponentially with cultivation time in the logarithmic growth phase and then reached a plateau in the stationary phase. The *S. avermitilis* after 20 pulses of 5 and 10 kV/cm nsPEFs treatments both show similar growth curves with the control (*P* > 0.05). However, since the 36^th^ hour, the OD_450_ readings of the *S. avermitilis* treated with 20 pulses of nsPEFs at 15 kV/cm presented significantly higher values than that of the control, suggesting that this parameter group of nsPEFs treatment could promote cell growth of *S. avermitilis*, while the lower ones had little effect on its cell growth. From these results, we believe that the nsPEFs can effectively regulate the cell growth of *S. avermitilis* and provides a feasible way to improve the yield of avermectin through optimizing the nsPEFs parameters.

### Effect of nsPEFs on avermectin production

It has been realized that the avermectin complexes have an ultraviolet absorbance peak at 245 nm^28^. Also, Gao Hong *et al*. reported that the accuracy and repeatability of UV spectrophotometry for quantifying avermectin was same as that of high performance liquid chromatography (HPLC)^29^. Thus, in this study, the yields of total avermectins were detected by OD readings at 245 nm (OD_245_) with a Rapid UV/Vis spectrometer. A calibration curve was obtained with commercial avermectin standards in the concentrations ranging from 6.4 to 64.0 *μ*g/ml (Fig. 5c). As shown in Fig. 5b, the avermectin production of control slightly increased and reached a plateauat 10.7 μg/ml on day 7. For the *S. avermitilis* treated by nsPEFs with 5 kV/cm, the avermectin production increased from 9.3 to 13.8 μg/ml in the first 7 days, and then decreased to 11.4 μg/ml on day 9. However, for those with 10 and 15 kV/cm nsPEFs treatments, the fermentation period of *S. avermitilis* to reach the highest avermectin production was shorten from 7 days to 5 days as compared to that of control and with 5 kV/cm nsPEFs treatment, and the avermectin production rose to 12.7 and 15.2 μg/ml, respectively, markedly higher than that of the control (10.7 μg/ml). It should be pointed out that by recalling the results in the last section, the nsPEF treatment at 15 kV/cm could raise the efficiency of avermectin fermentation not only by reducing the fermentation period, but increasing the avermectin production as well, which may result from the enhancement of cell growth. More interestingly, even though the lower intensity treatment (5 and 10 kV/cm) produced no significant proliferation effect on cell growth of *S. avermitilis* (Fig. 5a), it promoted avermectin production in the long term; especially at 10 kV/cm nsPEFs treatment the fermentation period was much reduced. Therefore, it could be deduced that the contributions of nsPEFs to the improvement of avermectin production may resulted from two ways: enhancing the cell growth of *S. avermitilis* and regulating the avermectin biosynthesis directly.

### Effect of nsPEFs on the colony appearance of *S. avermitilis* strains

**Figure 6:**
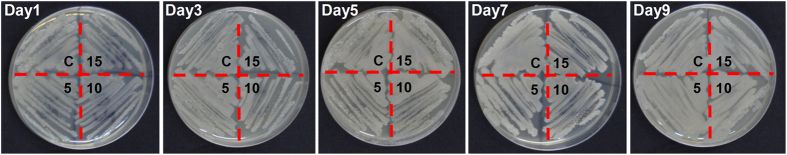
Images of *S. avermitilis* strains without (control) and with nsPEFs treatment grown on YMS agar during 9-day fermentation. C on the plate represents the *S. avermitilis* spores without any nsPEFs treatment (control); 5, 10 and 15, respectively, represent the *S. avermitilis* spores treated by nsPEFs with 5, 10, 15 kV/cm intensities. All pictures were obtained after the plate had been cultured for 3 days at 28 °C.

Generally, morphology and color are two primary indicators to assess bacterial colony appearance. Thus, to determine whether nsPEFs could alter colony appearance, the *S. avermitilis* spores of control and treated with nsPEFs were cultivated on the solid YMS medium on day 1, 3, 5, 7 and 9 during fermentation. As shown in Fig. 6, there were no visible differences in morphology and colors between the colonies developed from the control and treated spores, indicating that the electric field intensities of 5, 10 and 15 kV/cm by nsPEFs treatment could not cause any effect on the colony appearance of *S. avermitilis*. It is well known that varied parameters of nsPEFs, such as duration, number of pulses and intensity, could lead to different biological processes. Intense nsPEFs treatments with high field strength could induce apoptosis in human and mouse tumor cells^30^,^31^,^32^,^33^, bacterial inactivation with irreversible nanopores formation^34^, and DNA damage^35^. While, mild nsPEFs treatments with low field strength could activate intracellular signaling pathways^26^,^36^,^37^,^38^, improve the growth of *Arabidopsis thaliana* and *Haloxylon ammodendron* seeds, as well as enhance the cell proliferation^23^,^24^,^25^,^26^,^38^. Therefore, it is speculated that mild nsPEFs treatment in this study may not lead to the mutation of *S. avermitilis*. This presents a contrast result to the report from Yang *et al*., which suggested that *S. avermitilis* strains with different colony appearances giving various morphologies and colors were generated by atmospheric pressure glow discharge. High concentrations of chemically reactive species generated by plasma could destroy the mononucleotides’ phosphates group in cells and accordingly resulted in a strong mutagenic effect on *S. avermitilis*^5^. Furthermore, to determine whether the mild nsPEFs treatments used in this study could produce *S. avermitilis* mutants, it is need be further studied by combining with molecular biotechnology such as various omics technologies for comparative analysis of nsPEFs-treated strains and the control strains in the future.

### Evaluation of physicochemical properties of nsPEFs-treated solution

**Figure 7:**
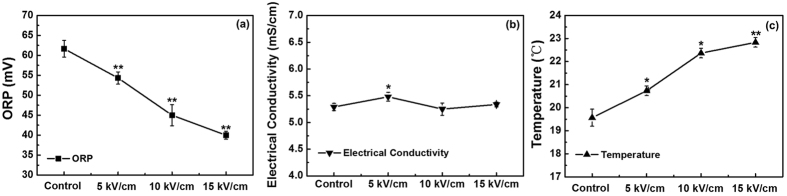
The values of (**a**) ORP, (**b**) electrical conductivity and (**c**) temperature of PBS without (control) and with nsPEFs treatment. Values are the mean ± standard deviation of three independent biological replicate experiments (n = 3). Statistical significance of differences between nsPEFs-treated and control PBS is indicated with *when *P* < 0.05, **when *P* < 0.01.

The Oxidation-Reduction Potential (ORP), temperature and electrical conductivity of the PBS solutions without nsPEFs treatment (control) and with 5, 10 and 15 kV/cm nsPEFs treatment were all immediately measured to evaluate their physical and chemical properties. As shown in Fig. 7a, the ORP decreased almost linearly with the pulsed electric filed intensity, changing from 61 to 40 mV, which was consistent with our previous work^25^. Recently, some studies proposed an economically competitive strategy based on ORP for improving fermentation efficiency, which suggest that ORP could serve as an index to evaluate the antibiotic production^39^,^40^,^41^. It is well known that the fermentation liquid of microorganism is generally not always in the redox equilibrium state in the fermentation process. The microorganisms absorb nutrients from the culture medium to obtain energy for cell growth and product synthesis by connecting the internal oxidation reduction reaction and intracellular metabolic processes. Some researchers have showed that ORP could regulate the microbial fermentation efficiency mainly by influencing the amount of dissolved oxygen level in the fermentation liquid. Moreover, ORP has a close relationship with the ethanol output in yeast cell^42^,^43^. On one hand, the trace amount of dissolved oxygen improved the cellular activity, accordingly increasing tolerance to ethanol. On the other hand, high levels of dissolved oxygen inhibited the metabolism of glycolytic pathway by acting as an electron acceptor substituted acetaldehyde, directly reducing the ethanol production. Furthermore, Lin *et al*. reported that ORP had a better relevance with the production of clavulanic acid than dissolved oxygen, which could increase the yield of carat clavulanic acid by 96%^44^. As a consequence, the decreased ORP has a close relationship with the improved fermentation efficiency of avermectin caused by nsPEFs. Furthermore, the temperature of PBS solution increased modestly with the pulsed electric field intensity (Fig. 7c). Accompanying with the 5, 10 and 15 kV/cm nsPEFs treatment, the temperature rose with the increasing electric field strengths, reaching approximately 20.7, 22.4 and 22.8 °C, respectively, which was similar to the results reported by Fox *et al*.^45^ It is well known that although PEF is intended to be a non-thermal technique, a temperature rise is present due to the electric current flowing in the liquid (ohmic heating), where the pulsed electric field energy input is transformed into heat^45^,^46^,^47^. Moreover, temperature is an important basic abiotic factors affecting the bacterial production and growth. Lewis *et al*. reported that the growth rate of *Achnanthes longipes* increased significantly (P < 0.0001) with increasing temperature until the maximum growth rate was reached at 26 °C, above which growth rate dropped sharply^48^. The suitable temperature for the cell growth of *S. avermitilis* is 28 °C in this study, below which the increasing temperature could improve the cell growth rate. Hence, there is a possibility that the increased temperature induced by nsPEFs is closely related with the enhanced cell growth of *S. avermitilis*. In Fig. 7b, the electrical conductivity of nsPEFs-treated PBS was almost similar with the control (around 5.25), indicating that electrical conductivity have no significant contribution to the cell growth and avermectin production of *S. avermitilis*.

### Effect of nsPEFs on expression of *aveR, malE* and *σ* ^25^

The entire genome sequence of *S. avermitilis*, including that of the gene cluster for the biosynthesis of avermectin, has already been determined^49^,^50^, and fundamental studies on the biosynthetic pathway of avermectins and their regulatory mechanism have also been conducted^2^,^51^. Some studies on functional genes and global regulatory genes, as well as on gene manipulation of pertaining to the biosynthesis of avermectins in *S. avermitilis* have been carried out^51^. In this study, three important genes, *aveR, malE* and *σ*^25^, associated with avermectin production of *S. avermitilis*, were analyzed by real-time RT-PCR tests. The avermectin gene cluster spans a distance of 82 kb and has a putative pathway-specific regulatory gene, *aveR*^49^, which encodes a pathway-specific activator essential for avermectin biosynthesis as well as a negative regulator for oligomycin biosynthesis^52^. Moreover, *malEFG-a* encodes proteins involved in the maltose transport system, and plays an essential role in avermectins production in *S. avermitilis*. Overexpression of *malEFG-a* in *S. avermitilis* enhanced the utilization rate of starch, resulting in increased antibiotic production and reduced fermentation periods^27^. In addition, *σ*^25^ is an extracytoplasmic function (ECF) σ factor in *S. avermitilis* that plays a differential regulatory role in avermectin and oligomycin biosynthesis. Gene deletion, complementation, and overexpression experiments showed that *σ*^25^ could indirectly inhibit avermectins production by affecting the transcription of the pathway-specific activator gene *aveR*^53^.

**Figure 8:**
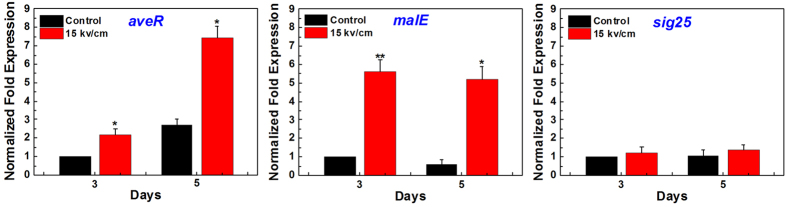
The real-time RT-PCR analysis of *aveR, malE* and *σ*^25^ transcript levels in *S. avermitilis* without (control) and with 15 kV/cm nsPEFs treatment performed on total RNA isolated on day 3 and day 5 during fermentation. The results are reported as the relative expression of *aveR, malE* and *σ*^*25*^ transcripts with respect to *hrdB* mRNA at the corresponding time points. The relative values for the expression of each gene on day 3 in control were defined as 1. Values are the mean ± standard deviation of three independent biological replicate experiments (n = 3). Statistical significance of differences between the nsPEFs-treated and control cells is indicated with *when *P* < 0.05, **when *P* < 0.01.

The result of Fig. 8a,b shows that the transcript levels of *aveR* and *malE* were significantly higher for the *S. avermitilis* strains treated by nsPEFs at 15 kV/cm than that of control on day 3 and day 5, indicating that nsPEFs could enhance the expression of *aveR* and *malE*. While, the transcript level of *σ*^25^ was almost the same without difference between the nsPEFs-treated group and the control (*P* > 0.05), suggesting that nsPEFs has no effect on the expression of *σ*^25^ during the fermentation process (Fig. 8c).

It was generally accepted that nsPEFs provoke a remarkable increase in cytoplasmic Ca^2+^, which presumably arises from the cumulative effects of nanopore formation and direct actions of nsPEFs on intracellular Ca^2+^ stores and various Ca^2+^ channels in the cell membrane^20^,^54^,^55^,^56^. Calcium and calcium-binding proteins including those resembling calmodulin are implicated in numerous diverse processes in bacteria including chemotaxis, sporulation, virulence, the transport of sugars and proteins, phosphorylation, heat shock, the initiation of DNA replication, septation, nucleoid structure, nuclease activity and recombination, the stability of envelope, and phospholipid synthesis and configuration^57^. Moreover, calcium ions could also serve as second messengers and provide important upstream signals for cellular mechanisms to occur such as proliferation, differentiation and apoptosis^58^. Therefore, recently, many researchers have started to pay attention on the specific signaling events provoked in response to Ca^2+^ mobilization by nsPEFs. K. Morotomi-Yano *et al*. provided experimental evidence that nsPEFs-induced Ca^2+^ mobilization directly affects the intracellular signal pathway involving calcium/calmodulin-dependent protein kinase kinase (CaMKK) and AMP-activated protein kinase (AMPK) in human cells^59^. G. P. Tolstykh *et al*. reported that exposing cells to nanosecond pulsed electric fields causes a rapid increase in intracellular calcium, enabling a pathway that activates protein kinase C (PKC) for various physiological functions, including hormone secretion, action potentials (AP) propagation, and muscle contraction^60^. Kun Zhang *et al*. also reported that nsPEFs could enhance the proliferation and dedifferentiation of chondrocytes via activation of the wnt/β-catenin signaling pathways accompanied by the bursts of intracellular calcium^26^.

Taken together, it is speculated that nsPEFs may induce calcium ion influx in *S. avermitilis* cells, the extent of which depends on nsPEFs dosage, set off a cascade of signaling events that dictate cellular response and may ultimately elevate the expression of *aveR* and *malE*, consequently resulting in the raising of avermectins production in *S. avermitilis*. Additionally, given the fact that the avermectins biosynthesis in *S. avermitilis* is too complex to understand clearly, future study will investigate the effects of nsPEFs on the complex metabolic pathways and global regulation networks of avermectins biosynthesis in *S. avermitilis* via molecular biotechnologies such as genomics, proteomics and metabolomics, which will eventually contribute to the creation of a powerful *S. avermitilis* strain for avermectins production.

In conclusion, the results in this study show that nsPEFs can significantly improve avermectins production by prompting cell growth and enhancing expression of gene associated closely with avermectin biosynthesis in *S. avermitilis*. As an alternative strategy, nsPEF could provide a potentially feasible way in avermectins fermentation.

## Methods

### Strain, media and culture conditions

The strain used in this work was *S. avermitilis* ATCC31267 (wild-type; avermectin-producer)^61^, which was grown at 28 °C on a solid YMS (yeast extract–malt extract–starch) medium for sporulation^62^. The seed medium for avermectin fermentation was composed of 30 g soluble starch, 4 g yeast extract, 2 g soya peptone, and 10 mg CoCl_2_·6H_2_O per liter H_2_O. The fermentation medium for avermectin production consisted of 70 g soluble starch, 16 g yeast power, 0.5 g K_2_HPO_4_·3H_2_O, 0.5 g MgSO_4_·7H_2_O, 4 g KCl, 10 mg CoCl_2_·6H_2_O, and 2 g CaCO_3_ per liter H_2_O^63^. The soluble FM-II (fermentation medium II) used to cultivate mycelia for growth and RT-PCR analysis was composed of 50 g soluble starch, 12 g yeast extract, 0.5 g MgSO_4_·7H_2_O, 4 g KCl, 5 mg CoCl_2_·6H_2_O, and 0.5 g K_2_HPO_4_·3H_2_O per liter H_2_O^52^. All the media were autoclaved at 121 °C for 20 min.

### Evaluation of cell viability of *S. avermitilis* after nsPEFs treatment

The survival rate of *S. avermitilis* spore under different operation conditions was evaluated based on the following equation:


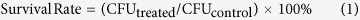


where^64^ CFU_control_ is the total colony count of the sample without nsPEFs treatment, and CFU_treated_ the total colony count after nsPEFs treatment. All the colony numbers were obtained by the colony forming units (CFU) assay on solid YMS medium.

### Measurement of cell growth of *S. avermitilis*

Soluble FM-II was used to measure the cell growth of *S. avermitilis*. The nsPEFs-treated spores were collected, rinsed twice with PBS and resuspended in 1 ml PBS. Then, 1 ml spore suspensions were cultured in 250-ml Erlenmeyer flasks containing 100 ml soluble FM-II for 11 days at 28 °C on a rotary shaker (180 rpm). The optical density readings at 450 nm (OD_450_) of *S. avermitilis* cells on a SPECTROstar Omega absorbance plate reader with a Rapid UV/Vis spectrometer (BMG, Germany) measured at different time points were used as the indication of biomass concentrations^65^.

### Morphology characterization of *S. avermitilis*

To evaluate the integrity of bacterial cells, the changes in the surface morphology of *S. avermitilis* cells after nsPEFs exposure were observed by scanning electron micrograph (SEM). After treatment, *S. avermitilis* cells were fixed with 2.5% glutaraldehyde in 0.1 M PBS (pH 7.2) at 4 °C overnight, and then dehydrated by a series of increasing ethanol (20, 40, 60, 80 and 100% v/v ethanol in water; 15 min in each solution). Subsequently, they were split longitudinally, dried naturally, fixed and sputter-coated with gold-palladium for examining with SEM (S-4800, HITACHI, Japan).

Moreover, the images of *S. avermitilis* spores of control and nsPEFs treatment grown on the solid YMS medium on day 1, 3, 5, 7 and 9 during fermentation were obtained after the plate had been cultured for 3 days at 28 °C.

### Fermentation and analysis of avermectin production

After the nsPEFs-treated *S. avermitilis* cultured in the fermentation medium for 9 days (mentioned above), 5 ml of the fermentation broth was sampled on day 1, 3, 5, 7 and 9 from every flask and centrifuged at 5000 g for 5 min. The precipitate was collected, and 2 ml acetone was added and shaken for 30 min. Subsequently, 3 ml ethyl acetate was added to the mixture and shaken for an additional 30 min. After the mixture was left standing for 10 min, 50 μl of the supernatant was then diluted with 2 ml methanol. The yields of total avermectins were detected by OD readings at 245 nm (OD_245_) with a Rapid UV/Vis spectrometer^29^, using authentic samples of avermectin as internal standards.

### Physicochemical properties measurement

Oxidation-reduction potential (ORP), electrical conductivity, and temperature of PBS were immediately measured after nsPEFs treatment. ORP and temperature were measured by a multimeter pH & Redox (Mettler-Toledo, Switzerland). And electrical conductivity was measured with electric conductivity meter (DDB-303A).

### RNA preparation and real-time RT-PCR analysis

After grown in soluble FM-II for 3 and 5 days, mycelia of *S. avermitilis* were collected, flash-frozen in liquid nitrogen, and ground into fine powder. Total RNA was isolated from the ground mycelial paste using Trizol reagent (Invitrogen, USA) according to the manufacturer’s instructions, and the quality of RNAs were assessed using a NanoDrop 2000 spectrophotometer (Thermo Scientific, USA). Then, the sample was treated with DNase I to remove the contaminating chromosomal DNA. The treated RNA sample (2 μg) was reverse transcribed using M-MLV (RNase H^−^, TaKaRa), random hexamers (25 μM), and a dNTP mixture (10 mM each). Real-time RT-PCR analysis was performed to determine the transcription levels of *aveR, malE* and *σ*^25^ using the obtained cDNA as template and with the gene-specific primers listed below: for *aveR*, 5′-CCGCGACTTCCTCACCG-3′ (sense) and 5′-GGACTCGCTCAGCAG-3′ (antisense); for *malE*, 5′-TACGCCCACATCCAGGACG-3′ (sense) and 5′-GTCGGCAGCG TGGAGTTC-3′ (antisense); for *σ*^25^, 5′-CTGCAGAGCGCGCTGTTC-3′ (sense) and 5′- GGTTGGTCATGGTGCGACG-3′ (antisense); for *hrdB*, 5′-TACTGCGCAGCCTCAACCAG-3′ (sense) and 5′-GCCGATCTGCTTGAGGTAGTC-3′ (antisense). The RT-PCR experiments were performed using FastStart Universal SYBR Green Master (ROX; Roche, USA) with analysis by an ABI 7900HI Sequence Detection System (Applied Biosystems, USA) using optical-grade 96-well plates. Template cDNA, 10 μl SYBR Green Master, and forward and reverse primers (each 300 nM) were mixed in each reaction system (total volume 20 μl). The PCR protocol consisted of 95 °C for 10 min, 40 cycles of 95 °C for 10 s, and 60 °C for 30 s with a single fluorescence measurement. For semi-quantitative analysis, transcript of *aveR, malE* and *σ*^25^ was normalized to that of *hrdB*, which encodes the major sigma factor in Streptomyces, was used as positive control for RT-PCR assay. RT-PCR without the initial reverse transcription step was carried out as a negative control for all analyses to confirm the absence of DNA contamination. Data were verified in three independent experiments.

### Statistical analysis

All data were obtained from three independent biological replicate experiments (n = 3). The values from all experiments were expresses as the mean ± standard deviation (SD). Statistical analysis was performed using SPSS statistical package 17.0 (SPSS Inc., USA). An analysis of variance (ANOVA) was conducted to compare the effects of different nsPEFs treatments on the avermectin production during formation process, and significant differences between the mean values were identified by the Student-Newman-Keul’s multiple range test with a confidence level at *P* ≤ 0.05. Moreover, the paired-sample t-test was applied to evaluate the statistical significance of differences between the nsPEFs-treated and control groups on the cell viability, cell growth and gene expression of *S. avermitilis*, as well as the physicochemical properties (ORP, electrical conductivity, pH and temperature) of PBS. The difference is expressed as *P < 0.05, **P < 0.01, and ***P < 0.001.

## Additional Information

**How to cite this article**: Guo, J. *et al*. Raising the avermectins production in *Streptomyces avermitilis* by utilizing nanosecond pulsed electric fields (nsPEFs). *Sci. Rep.* **6**, 25949; doi: 10.1038/srep25949 (2016).

## References

[b1] Burg R. W. . Avermectins, new family of potent anthelmintic agents: producing organism and fermentation. Antimicrob. Agents Ch. 15, 361–367 (1979). 10.1128/aac.15.3.361 PMC352666 464561

[b2] Ikeda H. & Omura S. Avermectin biosynthesis. Chem. Rev. 97, 2591–2610 (1997). 11851473 10.1021/cr960023p

[b3] Yoon Y. J. . Avermectin: biochemical and molecular basis of its biosynthesis and regulation. Appl. Microbiol. Biot. 63, 626–634 (2004). 10.1007/s00253-003-1491-4 14689246

[b4] Wang S. Y. . Mutagenesis, screening and industrialization of high avermectin B1a producing strains from *Streptomyces avermitilis* irradiated by 12C 6+ ion beam. Adv. Mater. Mater. 339, 652–655 (2011).

[b5] Wang L. . Novel mutation breeding method for *Streptomyces avermitilis* using an atmospheric pressure glow discharge plasma. J. Appl. Microbiol. 108, 851–858 (2010). 19735332 10.1111/j.1365-2672.2009.04483.x

[b6] Stutzman-Engwall K. . Engineering the *aveC* gene to enhance the ratio of doramectin to its CHC-B2 analogue produced in *Streptomyces avermitilis*. Biotechnol. Bioeng. 82, 359–369 (2003). 12599263 10.1002/bit.10578

[b7] Meng L. . Effects of maltose transport related gene expression on avermectins production in *Streptomyces avermitilis*. J. Agr. Sci. Tech. 16, 71–75 (2014).

[b8] Pfefferle C. . Improved secondary metabolite production in the genus streptosporangium by optimization of the fermentation conditions. J. Biotechnol. 80, 135–142 (2000). 10908794 10.1016/s0168-1656(00)00249-2

[b9] Cimburková E. . Nitrogen regulation of avermectins biosynthesis in *Streptomyces avermitilis* in a chemically defined medium. J. Basic Microb. 28, 491–499 (2007).

[b10] Lu Y. . Characteristics of hydrogen production of an *Enterobacter aerogenes* mutant generated by a new atmospheric and room temperature plasma (ARTP). Biochem. Eng. J. 55, 17–22 (2011).

[b11] Ganusov V. V., Bril’kov A. V. & Pechurkin N. S. Mathematical Modeling of Population Dynamics of Unstable Plasmid-bearing Bacterial Strains under Continuous Cultivation in a Chemostat. Biophysics 45, 881–887 (2000). 11094722

[b12] Cheng K. K., Liu H. J. & Liu D. H. Multiple growth inhibition of *Klebsiella pneumoniae* in 1,3-propanediol fermentation. Biotechnol. Lett. 27, 19–22 (2005). 15685414 10.1007/s10529-004-6308-8

[b13] Louise C. . Nanosecond electric pulse effects on gene expression. J. Membrane Biol. 246, 851–859 (2013). 23831956 10.1007/s00232-013-9579-y PMC3825134

[b14] Rems L. . Cell electrofusion using nanosecond electric pulses. Sci. Rep. 3, 1119–1120 (2013). 24287643 10.1038/srep03382 PMC3843160

[b15] Ren W. & Beebe S. J. An apoptosis targeted stimulus with nanosecond pulsed electric fields (nsPEFs) in E4 squamous cell carcinoma. Apoptosis 16, 359–369 (2011). 21213047 10.1007/s10495-010-0572-y PMC3066400

[b16] Wang J. . Synergistic effects of nanosecond pulsed electric fields combined with low concentration of gemcitabine on human oral squamous cell carcinoma *in vitro*. Plos One 7, 4A–4 (2012). 10.1371/journal.pone.0043213 PMC3426536 22927951

[b17] Qi W. . Synergistic effect of nanosecond pulsed electric field combined with low-dose of pingyangmycin on salivary adenoid cystic carcinoma. Oncol. Rep. 31, 2220–2228 (2014). 24604118 10.3892/or.2014.3063

[b18] Amiali M. . Synergistic effect of temperature and pulsed electric field on inactivation of Escherichia coli O157:H7 and Salmonella enteritidis in liquid egg yolk. J. Food Eng. 79, 689–694 (2007).

[b19] Zhang J. . Nanosecond pulse electric field (nanopulse): A novel non-ligand agonist for platelet activation. Arch. Biochem. Biophys. 471, 240–248 (2008). 18177729 10.1016/j.abb.2007.12.009

[b20] Vernier P. T. . Calcium burstsinduced by nanosecond electric pulses. Biochem. Biophys. Res. Co. 310, 286–295 (2003). 10.1016/j.bbrc.2003.08.140 14521908

[b21] Baldwin W. H. . Membrane permeability and cell survival after nanosecond pulsed-electric-field exposure—significance of exposure-media composition. IEEE T. Plasma Sci. 38, 2948–2953 (2010).

[b22] Schoenbach K. H. . Ultrashort electrical pulses open a new gateway into biological cells. P. IEEE 92, 1122–1137 (2004).

[b23] Eing C. . Effects of nanosecond pulsed electric field exposure on arabidopsis thaliana. IEEE T. Dielect. El. In. 16, 1322–1328 (2009).

[b24] Songnuan W. & Kirawanich P. Early growth effects on Arabidopsis thaliana by seed exposure of nanosecond pulsed electric field. J. Eelectrostat. 70, 445–450 (2012).

[b25] Su B. . Early Growth Effects of Nanosecond Pulsed Electric Field (nsPEFs) Exposure on *Haloxylon ammodendron*. Plasma Process. Polym. 12, 372–379 (2015).

[b26] Zhang K., Guo J., Ge Z. & Zhang J. Nanosecond Pulsed Electric Fields (nsPEFs) Regulate Phenotypes of Chondrocytes through Wnt/β-catenin Signaling Pathway. Sci. Rep. 4, 1–8 (2014). 10.1038/srep05836 PMC5376156 25060711

[b27] Li M. . Enhancement of avermectin and ivermectin production by overexpression of the maltose ATP-binding cassette transporter in *Streptomyces avermitilis*. Bioresource Technol. 101, 9228–9235 (2010). 10.1016/j.biortech.2010.06.132 20655739

[b28] Miller T. W. . Avermectins, new family of potent anthelmintic agents: isolation and chromatographic properties. Antimicrob. Agents Ch. 15, 368–371 (1979). 10.1128/aac.15.3.368 PMC352667 464562

[b29] Gao H. . Identification of avermectin-high-producing strains by high-throughput screening methods. Appl. Microbiol. Biot. 85, 1219–1225 (2010). 10.1007/s00253-009-2345-5 19957083

[b30] Beebe S. J., Fox P. M., Rec L. J., Willis E. L. & Schoenbach K. H. Nanosecond, highintensity pulsed electric fields induce apoptosis in human cells. FASEB J. 17, 1493–1495 (2003). 12824299 10.1096/fj.02-0859fje

[b31] Garon E. B. . *In vitro* and *in vivo* evaluation and a case report of intense nanosecond pulsed electric field as a local therapy for human malignancies. Int. J. Cancer 121, 675–682 (2007). 17417774 10.1002/ijc.22723

[b32] Nuccitelli R. . A new pulsed electric field therapy for melanoma disrupts the tumor’s blood supply and causes complete remission without recurrence. Int. J. Cancer 125, 438–445 (2009). 19408306 10.1002/ijc.24345 PMC2731679

[b33] Ford W. E., Ren W., Blackmore P. F., Schoenbach K. H. & Beebe S. J. Nanosecond pulsed electric fields stimulate apoptosis without release of pro-apoptotic factors from mitochondria in B16f10 melanoma. Arch. Biochem. Biophys. 497, 82–89 (2010). 20346344 10.1016/j.abb.2010.03.008

[b34] Ulmer H. M., Heinz V., Ga¨nzle M. G., Knorr D. & Vogel R. F. Effects of pulsed electric fields on inactivation and metabolic activity of *Lactobacillus plantarum* in model beer. J. Appl. Microbiol. 93, 326–335 (2002). 12147082 10.1046/j.1365-2672.2002.01699.x

[b35] Stacey M. . Differential effects in cells exposed to ultra-short, high intensity electric fields: cell survival, DNA damage, and cell cycle analysis. Mutat. Res. 542, 65–75 (2003). 14644355 10.1016/j.mrgentox.2003.08.006

[b36] Morotomi-Yano K., Uemura Y., Katsuki S., Akiyama H. & Yano K. Activation of the JNK pathway by nanosecond pulsed electric fields. Biochem. Bioph. Res. Co. 408, 471–476 (2011). 10.1016/j.bbrc.2011.04.056 21521634

[b37] Morotomi-Yano K., Akiyama H. & Yano K. Nanosecond pulsed electric fields activate MAPK pathways in human cells. Arch. Biochem. Biophys. 515, 99–106 (2011). 21933660 10.1016/j.abb.2011.09.002

[b38] Morotomi-Yano K., Oyadomari S., Akiyama H. & Yano K. Nanosecond pulsed electric fields act as a novel cellular stress that induces translational suppression accompanied by eIF2alpha phosphorylation and 4E-BP1 dephosphorylation. Exp. Cell Res. 318, 1733–1744 (2012). 22652449 10.1016/j.yexcr.2012.04.016

[b39] Song J. . Effects of pH and ORP on microbial ecology and kinetics for hydrogen production in continuously dark fermentation. Bioresource Technol. 102, 10875–10880 (2011). 10.1016/j.biortech.2011.09.024 21978625

[b40] Du C. . Use of oxidoreduction potential as an indicator to regulate 1, 3-propanediol fermentation by *Klebsiella pneumoniae*. Appl. Microbiol. Biot. 69, 554–563 (2006). 10.1007/s00253-005-0001-2 16021488

[b41] Sheu D. C. . Production of xylitol from *Candida tropicalis* by using an oxidation-reduction potential-stat controlled fermentation. Biotechnol. Lett. 25, 2065–2069 (2003). 14969410

[b42] Burmaster Brian, M. inventors. Ethanol fermentation using oxidation reduction potential. United States patent US 7,078,201. 2006 Jul 18.

[b43] Liu C. G., Wang N., Lin Y. H. & Bai F. W. Very high gravity ethanol fermentation by flocculating yeast under redox potential-controlled conditions. Biotechnol. Biofuels 5, 1–7 (2012). 22917193 10.1186/1754-6834-5-61 PMC3494525

[b44] Lin Y. H. . Using redox potential to detect microbial activities during clavulanic acid biosynthesis in *Streptomyces clavuligerus*. Biotechnol. Lett. 27, 1791–1795 (2005). 16314972 10.1007/s10529-005-3727-0

[b45] Fox M. B., Esveld D. C., Mastwijk H. & Boom R. M. Inactivation of *L. plantarum* in a PEF microreactor–the effect of pulse width and temperature on the inactivation. Innov. Food Sci. Emerg. 9, 101–108 (2008).

[b46] Lindgren M., Aronsson K., Galt S. & Ohlsson T. Simulation of the temperature increase in pulsed electric field (PEF) continuous flow treatment chambers. Innov. Food Sci. Emerg. 3, 233–245 (2002).

[b47] Jaeger H., Meneses N. & Knorr D. Impact of PEF treatment inhomogeneity such as electric field distribution, flow characteristics and temperature effects on the inactivation of *E. coli* and milk alkaline phosphatase. Innov. Food Sci. Emerg. 10, 470–480 (2009).

[b48] Lewis R. J., Johnson L. M. & Hoagland K. D. Effects of cell density, temperature, and light intensity on growth and stalk production in the biofouling diatom *Achnanthes longipes* (Bacillariophyceae). J. Phycol. 38, 1125–1131 (2002).

[b49] Ikeda H., Nonomiya T., Usami M., Ohta T. & Omura S. Organization of the biosynthetic gene cluster for the polyketide anthelmintic macrolide avermectin in *Streptomyces avermitilis*. P. Natl. Acad. Sci. USA 96, 9509–9514 (1999). 10.1073/pnas.96.17.9509 PMC22239 10449723

[b50] Ikeda H. . Complete genome sequence and comparative analysis of the industrial microorganism *Streptomyces avermitilis*. Nat. Biotechnol. 21, 526–531 (2003). 12692562 10.1038/nbt820

[b51] Ikeda H., Nonomiya T. & Omura S. Organization of biosynthetic gene cluster for avermectin in *Streptomyces avermitilis*: analysis of enzymatic domains in four polyketide synthases. J. Ind. Microbiol. Biotechnol. 27, 170–176 (2001). 11780788 10.1038/sj.jim.7000092

[b52] Guo J. . The pathway-specific regulator *AveR* from *Streptomyces avermitilis* positively regulates avermectin production while it negatively affects oligomycin biosynthesis. Mol. Genet. Genomics 283, 123–133 (2010). 20012992 10.1007/s00438-009-0502-2

[b53] Luo S. . An extracytoplasmic function sigma factor, *σ*^25^, differentially regulates avermectin and oligomycin biosynthesis in *Streptomyces avermitilis*. Appl. Microbiol. Biot. 98, 7097–7112 (2014). 10.1007/s00253-014-5759-7 24811406

[b54] White J. A., Blackmore P. F., Schoenbach K. H. & Beebe S. J. Stimulation of capacitative calcium entry in HL-60 cells by nanosecond pulsed electric fields. J. Biol. Chem. 279, 22964–22972 (2004). 15026420 10.1074/jbc.M311135200

[b55] Craviso G. L., Choe S., Chatterjee P., Chatterjee I. & Vernier P. T. Nanosecond electric pulses: a novel stimulus for triggering Ca^2+^ influx into chromaffin cells via voltage-gated Ca^2+^ channels. Cell. Mol. Neurobiol. 30, 1259–1265 (2010). 21080060 10.1007/s10571-010-9573-1 PMC11498812

[b56] Beier H. T., Roth C. C., Tolstykh G. P. & Ibey B. L. Resolving the spatial kinetics of electric pulse-induced ion release. Biochem. Biophys. Res. Co. 423, 863–866 (2012). 10.1016/j.bbrc.2012.06.055 22713455

[b57] Norris V. . Calcium in bacteria: a solution to which problem? Molecular Microbiology 5, 775–778 (1991). 1857203 10.1111/j.1365-2958.1991.tb00748.x

[b58] Nutt L. K. . Bax-mediated Ca^2+^ mobilization promotes cytochrome c release during apoptosis. J. Biol. Chem. 277, 20301–20308 (2002). 11909872 10.1074/jbc.M201604200

[b59] Morotomi-Yano K., Akiyama H. & Yano K. Nanosecond pulsed electric fields activate AMP-activated protein kinase: Implications for calcium-mediated activation of cellular signaling. Biochem. Biophys. Res. Co. 428, 371–375 (2012). 10.1016/j.bbrc.2012.10.061 23103546

[b60] Tolstykh G. P., Beier H. T., Thompson G. L., Roth C. C. & Ibey B. L. Nanosecond pulsed electric fields activate intracellular signaling pathways. Spienewsroom, doi: 10.1117/2.1201302.004736 (2013).

[b61] Hwang Y. S. . Optimization of transformation procedures in avermectin high-producing *Streptomyces avermitilis*. Biotechnol. Lett. 23, 457–462 (2001).

[b62] Ikeda H., Kotaki H., Tanaka H. & Omura S. Involvement of glucose catabolism in avermectin production by *Streptomyces avermitilis*. Antimicrob. Agents Ch. 32, 282–284 (1988). 10.1128/aac.32.2.282 PMC172155 3364948

[b63] Chen Z. . Enhancement and selective production of avermectin B by recombinants of *Streptomyces avermitilis* via intraspecific protoplast fusion. Chinese Sci. Bull. 52, 616–622 (2007).

[b64] Phornphisutthimas S. . Development of an intergeneric conjugal transfer system for rimocidin-producing *Streptomyces rimosus*. Lett. Appl. Microbiol. 50, 530–536 (2010). 20337930 10.1111/j.1472-765X.2010.02835.x

[b65] Hodgson D. A. Glucose repression of carbon source uptake and metabolism in *Streptomyces coelicolor* A3(2) and its perturbation in mutants resistant to 2-deoxyglucose. J. Gen. Microbiol. 128, 2417–2430 (1982).

